# Supplementary material for: Identification of photoperiod-induced specific miRNAs in the adrenal glands of Sunite sheep (Ovis aries)
Source: Front Vet Sci. 2022 Jul 22;9:888207. doi: 10.3389/fvets.2022.888207 (PMC9354845; doi:10.3389/fvets.2022.888207)
Supplement: Supplementary file 1 [file Data_Sheet_1.ZIP › Supplementary file/miRDeep2 file.zip!/miRDeep2 file/Know_Structure/SP42A2/oar-mir-382.pdf]

miRBase precursor : oar-mir-382
Total read count : 6028
oar-miR-382-5p read count : 2601
oar-miR-382-3p read count : 3416
remaining reads : 11

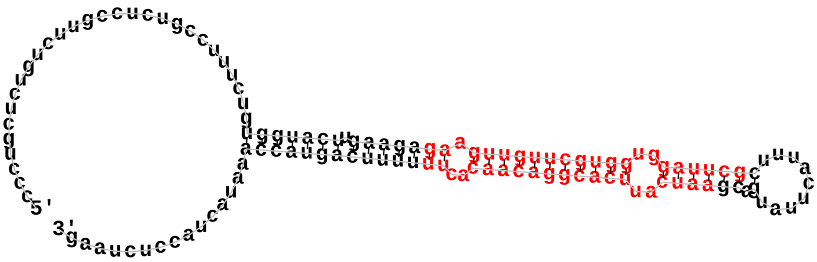

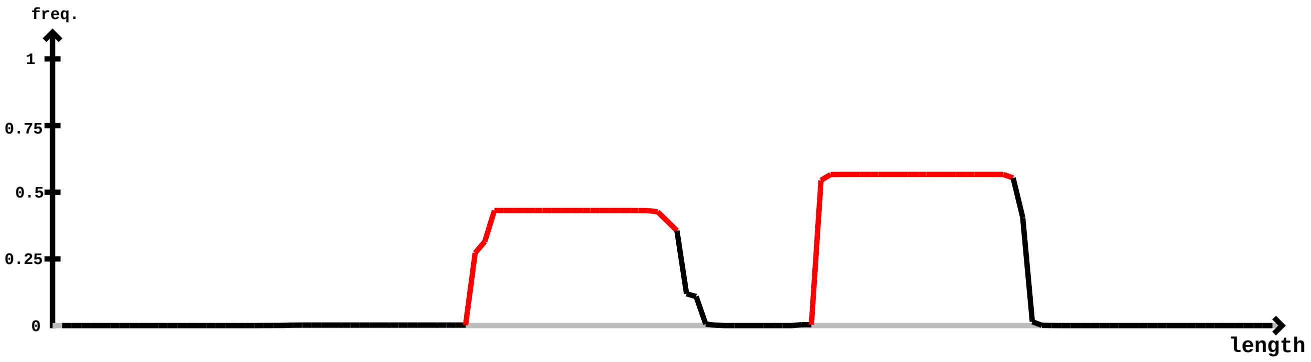

oar-miR-382-3p

oar-miR-382-5p

5'- cccugcucugucuugccucugccuuucugugguacuugaagagaaguuguucgugguggauucgcuuuacuuaugacgaaucauucacggacaacacuuuuuucaguaccaaauacuaccucuaag -3'	exp	
....((........))............(((((((.(((((((.(((((((((((..(((((((.........).))))))..)))))))))))..))))))))))))))................	reads	mm
....................gccuuucugugguacuugaaga..	1	0
.....................ccuuucugugguacuugaaga..	1	0
......................cuuucugugguacuugaaga..	1	0
.......................uuucugugguacuugaaga..	6	0
........................uucugugguacuugaaga..	1	0
........................uucugugguacuugaagaga..	1	0
....gaaguuguucguggugg...	1	0
....gaaguuguucguggugga..	1	0
....gaaguuguucguggugau...	27	0
....gaaguuguucgugguggauu..	199	0
....gaaguuguucgugguggauuc...	195	0
....gaaguuguucgugguggauucg..	1195	0
....gaaguuguucgugguggauucgc...	23	0
....gaaguuguucgugguggauucgcu..	1	0
....aaguuguucgugguggau...	1	0
....aaguuguucgugguggauu..	10	0
....aaguuguucgugguggauuc...	17	0
....aaguuguucgugguggauucg..	221	0
....aaguuguucgugguggauucgc...	3	0
....aaguuguucgugguggauucgcu..	3	0
....aaguuguucgugguggauucgcuu...	1	0
....aguuguucgugguggauu..	2	0
....aguuguucgugguggauuc...	2	0
....aguuguucgugguggauucg..	12	0
....aguuguucgugguggauucgc...	35	0
....aguuguucgugguggauucgcu..	620	0
....aguuguucgugguggauucgcuu...	23	0
....aguuguucgugguggauucgcuuu..	9	0
....cgaaucauucacggacaacacu............................	9	0
....cgaaucauucacggacaacacuu...........................	10	0
....cgaaucauucacggacaacacuuu..........................	1	0
....gaaucauucacggacaacacuu...........................	1	0
....aaucauucacggacaacac.............................	1	0
....aaucauucacggacaacacu............................	67	0

```
                                                                              oar-miR-382-3p
                                            oar-miR-382-5p
cccugcucugucuugccucugccuuucugugguacuugaagagaaguuguucgugguggauucgcuuuacuuaugacgaaucauucacggacaacacuuuuuucaguaccaaauacuaccucuaag

..............................................................................aaucauucacggacaacacuu...........................    870     0
..............................................................................aaucauucacggacaacacuuu..........................    2272    0
..............................................................................aaucauucacggacaacacuuuu.........................    50      0
..............................................................................aaucauucacggacaacacuuuuu........................    1       0
...............................................................................aucauucacggacaacacu............................    1       0
...............................................................................aucauucacggacaacacuu...........................    13      0
...............................................................................aucauucacggacaacacuuu..........................    84      0
...............................................................................aucauucacggacaacacuuuu.........................    31      0
...............................................................................aucauucacggacaacacuuuuu........................    3       0
................................................................................ucauucacggacaacacuuuu.........................    1       0
................................................................................ucauucacggacaacacuuuuuu.......................    1       0
```
